# Disease Activity Measures in Paediatric Rheumatic Diseases

**DOI:** 10.1155/2013/715352

**Published:** 2013-09-08

**Authors:** Nadia J. Luca, Brian M. Feldman

**Affiliations:** ^1^Division of Rheumatology, Department of Paediatrics, University of Toronto and The Hospital for Sick Children, 555 University Avenue, Toronto, ON, Canada M5G 1X8; ^2^Departments of Medicine, Institute of Health Policy Management and Evaluation and the Dalla Lana School of Public Health, University of Toronto, 155 College Street Toronto, ON, Canada M5T 3M7

## Abstract

Disease activity refers to potentially reversible aspects of a disease. Measurement of disease activity in paediatric rheumatic diseases is a critical component of patient care and clinical research. Disease activity measures are developed systematically, often involving consensus methods. To be useful, a disease activity measure must be feasible, valid, and interpretable. There are several challenges in quantifying disease activity in paediatric rheumatology; namely, the conditions are multidimensional, the level of activity must be valuated in the context of treatment being received, there is no gold standard for disease activity, and it is often difficult to incorporate the patient's perspective of their disease activity. To date, core sets of response variables are defined for juvenile idiopathic arthritis, juvenile systemic lupus erythematosus, and juvenile dermatomyositis, as well as definitions for improvement in response to therapy. Several specific absolute disease activity measures also exist for each condition. Further work is required to determine the optimal disease activity measures in paediatric rheumatology.

## 1. Introduction 

Measurement of a health state is the cornerstone of clinical practice and medical research. As stated in 1883 by Lord Kelvin, “when you can measure what you are speaking about, and express it in numbers, you know something about it; but when you cannot, your knowledge is of a meagre and unsatisfactory kind” [1]. When caring for children and youth with rheumatic diseases, standardized and validated definitions of disease activity, as well as of change in disease activity in response to treatment, are critical for making decisions in clinical practice as well as for conducting clinical research studies. Use of standardized measures can also facilitate comparisons between trials through meta-analyses.

Over the past several years, an international collaborative effort has been made to develop and validate measures of disease activity, treatment response, inactive disease, and flare of disease in several paediatric rheumatic conditions [2]. These measures are numerous, and several new ones are emerging on an annual basis. This paper will discuss the theoretical basis of disease activity measures, methods for their development and validation, and an overview of available measures within paediatric rheumatology. 

## 2. Definition of Disease Activity in Paediatric Rheumatic Diseases

In order to measure disease activity, it is necessary to define it and to identify its measurable components. “Disease activity” may be defined as aspects of a patient's disease that are potentially reversible [3–5]. This is in contrast to “disease damage,” which refers to the irreversible (i.e., due to scarring) manifestations of the disease or its treatment. Both disease activity and damage contribute to “disease severity,” which is the total effect of the disease on the individual and often relates to prognosis [5]. Disease activity may fluctuate widely during a patient's disease course. In the context of rheumatic diseases, a disease activity measure typically attempts to quantify the inflammatory process of the disease [3]. Consolaro et al. define disease activity in juvenile idiopathic arthritis (JIA) as measuring “signs and symptoms related to inflammation,” which “should separated from measures of disease severity, such as those that assess… structural damage” [6]. Evaluating disease activity may include measurement of biochemical markers of inflammation (e.g., erythrocyte sedimentation rate (ESR)), quantifying the amount of inflammatory tissue (e.g., number of swollen joints), and assessing the clinical consequences of inflammation (e.g., degree of morning stiffness). Generally, disease activity in most paediatric rheumatic diseases is multidimensional, and therefore this concept is measured in several domains [7]. For example, a measure of disease activity in juvenile dermatomyositis (JDM) incorporates muscle strength, skin disease, internal organ manifestations, and any other disease components in patients with JDM. Disease activity measures may take the form of observed values (e.g., muscle enzymes level), rating scales completed by health care providers, parent- or patient-reported outcomes (PROs), or any combination of these. When measuring disease activity, its impact on the biological, individual, and social perspectives of health is often considered, as described in the International Classification of Functioning, Disability, and Health (World Health Organization) [8, 9]. This system takes into account that disease activity interacts with internal and external factors to determine the levels of biological, physical, and social functioning. For example, a child with JIA who has swelling and reduced motion of a joint may have difficulty running and may not participate in gym class because of fear of injuring herself. Interventions can be aimed at each level: medical treatment to reduce joint inflammation, physiotherapy to improve running, and accommodations at school to allow the child to participate at their own pace. 

## 3. Challenges in Measuring Disease Activity in Paediatric Rheumatic Diseases

Given the multidimensionality of disease activity in paediatric rheumatic disease, a challenge exists when some components suggest that the disease is more active, while others suggest that it is less active. Thus there is appeal to a pooled and appropriately weighted composite index that can give one answer. For example, the American College of Rheumatology (ACR) core outcome variables for JIA were combined into a definition to dichotomously divide patients into those who improve by a clinically important amount and those who do not [10]. Variables in the core set include: (1) physician global assessment of disease activity, (2) parent/patient assessment of overall wellbeing, (3) functional ability, (4) number of joints with active arthritis, (5) number of joints with limited range of motion (ROM), and (6) ESR. Consensus methods were used to define improvement; for example, an ACR Pedi30 response requires ≥30% improvement from baseline in 3 of 6 variables, with ≤1 remaining variable worsening by >30%. Similar core sets and definitions for improvement have also been developed for JDM and juvenile systemic lupus erythematosus (JSLE) [11–14].

The ACR Pedi response criteria are defined relative to each patient's baseline parameters but do not enable the quantification of absolute disease activity or comparison of absolute responses amongst patients. For instance, these response criteria cannot distinguish between a patient who has improved by 30% starting with 30 active joints (still has 21 active joints) and one who started with 3 active joints (still has 2 active joints). This prompted the development of a scale that quantifies the absolute level of disease activity, the Juvenile Arthritis Disease Activity Score (JADAS), which includes 4 items: (1) physician global assessment of disease activity, (2) parent/patient global assessment of wellbeing, (3) number of active joints, and (4) ESR [6]. The JADAS is scored on a numerical scale, which allows patients to be compared directly. Absolute disease activity measures have also been developed for JSLE (e.g., European consensus lupus activity measurement (ECLAM), systemic lupus erythematosus disease activity index (SLEDAI)) and JDM (e.g., disease activity score (DAS), myositis disease activity assessment tool (MDAAT)) and are discussed further in Section 6.

An additional factor to consider is how to value clinical improvement in the context of the treatment regimen of the patient. For example, if one polyarticular JIA patient is managed on a nonsteroidal anti-inflammatory drug and achieves inactive disease, is his/her “disease activity” equivalent to another patient whose disease is quiescent while taking methotrexate and an anti-tumor necrosis factor alpha agent? Criteria for inactive disease and clinical remission in JIA do distinguish between patients who are on or off medical therapy. Wallace et al. define “inactive disease” as when a patient on medical therapy achieves the following criteria: (1) no joints with active arthritis, (2) no fever, rash, serositis, splenomegaly, or generalized lymphadenopathy, (3) no active uveitis, (4) normal ESR and/or C-reactive protein (CRP), and (5) physician global assessment indicates no disease activity [15]. If patients meet the inactive disease criteria for 6 continuous months while taking medication they are classified as being in “clinical remission on medication,” and if they meet the criteria for 12 continuous months off medication they are considered to be in “clinical remission off medication” [15]. Each of these health states is considered to be a distinct outcome. However, these criteria do not take into consideration the relative intensity of the treatment required to control the disease. 

Another significant challenge when measuring disease activity in pediatric rheumatic disease is that expert clinical judgment (i.e., opinion of a paediatric rheumatologist) is commonly used for validation of disease activity instruments [3], and this can be ambiguous and subjective. Thus, a limitation is that there is no truly objective “gold” standard with which to validate disease activity measures, although some promising biomarkers are being identified [16]. In addition, it is important that “circularity of reasoning” is avoided [17]. That is, if the group of clinicians that derives the criteria set is the same group that classifies disease activity, there will be a strong correlation between the criteria set and the so called “gold” standard. Lastly, the use of a physician-based measure alone for comparison entirely excludes the patient perspective of the disease burden.

Parent or patient global assessments are included in many paediatric rheumatic disease activity measures. Interestingly, studies show concordance with physician assessments of JIA disease activity in only about 40% of cases [18–20]. Sztajnbok et al. report that physicians tend to given higher global ratings compared to parents [18]. Conversely, parents gave scores of zero in only 65% of cases who met the Wallace definition of inactive disease in JIA (listed above) [19]. Parents are more likely to score higher disease activity if their child has a shorter disease duration, is taking second-line drug therapy, has increased reported pain, or has functional impairment. Alternatively, physicians consistently rate disease activity more highly than parents in the presence of any active joints [18, 19]. In JDM patients, Rider et al. found that physician and parent global ratings of disease activity were not collinear and that the nonredundancy may be the result of each evaluating different aspects of the disease [21]. This may also be because parents are often asked about overall “wellbeing” rather than specifically “disease activity.” Thus, the parent or patient perspective adds information on the impact of disease activity on overall health. 

For this reason, several new composite disease assessment measures have included more PROs. The juvenile arthritis parent assessment index (JAPAI) and the juvenile arthritis child assessment index (JACAI) include: (1) parent/child rating of overall wellbeing, (2) parent/child rating of pain intensity, (3) assessment of physical function, and (4) assessment of HRQOL [22]. Similarly, the juvenile arthritis multidimensional assessment report (JAMAR) incorporates parent- or patient-reported physical functioning, social participation, and HRQOL, among other items [23]. 

Although it can be challenging to validate PRO measures as they are inherently subjective, they provide rich information on the patient's perspective of their disease activity. However, it should be noted that paediatric rheumatologists developed these measures, and so they include items that health care providers judge as significant to patients but may not address all of the perceptions, values, and preferences important to patients. 

## 4. Development of Disease Activity Measures 

Measurement instruments are developed systematically, and the process is often iterative [7, 24, 25]. An approach for developing outcome measures in paediatric rheumatic diseases has been described in detail by Brunner and Ravelli [25]. Here we present a brief overview of the process (summarized in Table 1).

### 4.1. Purpose of the Measure

Along with defining what is to be measured and in which population, the purpose of the disease activity measure should be explicitly determined at the outset. Kirshner and Guyatt [45] describe three main purposes of health status measures. Firstly, “discriminative” measures are used to distinguish between individuals or groups with a certain attribute (e.g., criteria for inactive disease in JIA [15]). “Predictive” measures classify individuals into a set of predefined risk categories when a gold standard is available. This type of index is generally used as a screening or diagnostic instrument (e.g., dipstick proteinuria in lupus nephritis). Lastly, “evaluative” measures are used to assess the magnitude of longitudinal change in the dimension of interest (e.g., manual muscle testing (MMT) in JDM [46]). Disease activity measures typically fall into the evaluative category as they typically measure clinically significant changes in disease activity over time and in response to interventions. 

### 4.2. Item Generation and Reduction

The process of item generation creates a list of all possible items for inclusion, generally thorough the literature review and opinions from important stakeholders [47]. Ideally, patients and parents should be included in the process of measure development [48]. Item generation is followed by item reduction, with the goal of including all the important items but minimizing redundancy. Consensus methods have commonly been used for this phase of measure development (see below). Ideally, it is also important to determine the number of different aspects of disease activity, or “factors,” being measured. Principal component and factor analysis identify the number of factors within the scale and which of these explain the majority of the overall variance [26, 49]. At the item level, item-scale correlations and measures of internal consistency (Cronbach's alpha) can indicate which individual items within the factors may be redundant and possibly unnecessary [26].

### 4.3. Consensus Methods

In recent years, consensus methods such as Delphi surveys and nominal group techniques (NGTs) have commonly been used in the item generation and item reduction phases of measure development in paediatric rheumatology [50]. The definition of consensus should be stated a priori and utilized as criteria for termination of the process [51], although this is not always the case in practice [52]. The Delphi technique is an iterative multistage method that allows the use of anonymous written or online responses [53]. It involves a series of questionnaires, each of which is based on the results of the previous step, and the process stops when participants approach consensus. NGT utilizes a highly structured face-to-face meeting to gather information from relevant stakeholders [53]. Panelists rank, discuss, and then rerank a series of items related to the topic. The results are analyzed for agreement in prioritization, and generally 70–80% consensus is required [51]. These approaches have been used successfully to develop several disease activity measures in paediatric rheumatology such as the definition for improvement in JIA [10] and disease activity core set measures for JDM and JSLE [11].

### 4.4. Derivation Study

Once candidate sets of criteria are determined, they should be applied to a large and diverse group of patients with the disease from a variety of practices [17, 26]. A comparator group is chosen based on the intended use of the criteria and should represent patients from whom the criteria aim to distinguish. In order to avoid circularity of reasoning, the group of clinicians who create the list of candidate items should be separate from those who provide and classify the patients [17, 26]. The sample size required for derivation studies is approximately 100 per group [24].

### 4.5. Selection of Final Set of Items

The final set of items is often selected using statistical techniques such as comparing the sensitivity and specificity of candidate sets of items with receiver operating characteristic curve analysis. This was performed by Brunner et al. to determine the best definition for flare in patients with JIA [33]. Logistic regression may also be used to determine what variables best discriminate between more and less severely affected patients as was employed in the development of criteria for minimal disease activity (MDA) in JIA [42].

### 4.6. Assessment of Psychometric Properties

Prior to carrying out formal psychometric testing of a measurement tool, it should be pretested in a small group of users to ensure comprehensibility and relevance [47]. Evaluation of face and content validity ensures the items are reasonable and cover all the aspects of the disease. Feasibility should also be assessed, with consideration given to ease of use and minimal burden on the patient and health care provider. Without these characteristics, the tool is unlikely to be accepted by medical practitioners or adopted into clinical care. 

Once a tool is developed and piloted, its measurement properties must be evaluated in the target population. Most disease activity measures should be assessed with regards to their reliability, validity, responsiveness, and interpretability (Table 2) [28]. As a final step, measures should be applied to an independent sample to ensure external validation [24, 27].

## 5. Disease Activity Measures in JIA, JSLE, and JDM

A summary of the most commonly cited disease activity measures in JIA, JSLE, and JDM is presented in Table 3.

### 5.1. Core Response Variables in JIA, JSLE, and JDM

Composite disease activity measures comprising 5 or 6 core response variables (CRV) have been defined for each disease by consensus methodologies involving members of the ACR, PRINTO, and/or International Myositis Assessment and Clinical Studies (IMACS) groups, as mentioned above. Note that there are 2 slightly different core sets for JDM developed by PRINTO and IMACS (Table 3). The common components for each disease include physician and parent or patient global assessments, which use 10 cm visual analogue scales (VAS) to evaluate the extent of active inflammation based on all information available [21]. A score of zero indicates inactive disease and 10 indicates extremely high activity. In addition, the JIA and JDM core sets include a measure of physical function (most typically the childhood health assessment questionnaire (CHAQ), which has been validated in both populations [54, 55]). The CHAQ is divided into disability and discomfort indices which assess function in 8 areas (score 0–3) and pain intensity and overall wellbeing, respectively [54]. The JSLE and JDM core sets include a generic measure of HRQL that has been validated in both populations, the child health questionnaire (CHQ). The CHQ contains 14 domains to evaluate the physical, emotional, and social components of health and provides physical and psychosocial summary scores [56]. A notable challenge is that measures of physical function and HRQL within the core sets may reflect disease damage in addition to disease activity. The remaining CRV are specific to each disease and are further discussed in the sections below. 

Clinically meaningful improvement in the core set is often used in clinical research to define the primary endpoint. Criteria for improvement have been developed for each composite measure [10, 29–32], and a definition of disease flare has been proposed for JIA only [33]. As noted in Section 4, achieving improvement or flare is relative to the patient's baseline status, and thus the absolute degree of disease activity is different for each patient. 

### 5.2. Disease Activity Measures in JIA

In addition to the items mentioned above, the ACR core set also includes a measure of systemic inflammation (usually ESR or CRP), the number of joints with active arthritis (defined as joint effusion or limitation of motion accompanied by heat, pain, or tenderness), and the number of joints with limited ROM [10]. A systematic review of outcome measures in JIA compares the psychometric properties of the measures within the ACR core set, although it did not identify any validation studies of the composite core set itself [57]. The CHAQ has strong reliability and moderate correlations with other measures of disease activity but poor responsiveness. Active joint count and physician global assessment have the strongest correlations with disease activity and are most responsive to change.

The JADAS is a measure of absolute disease activity in JIA. It is similar to the core set in that it incorporates physician and parent/patient global assessment of disease activity, number of active joints, and ESR; however, it does not include functional status or number of joints with reduced ROM because these may be more reflective of disease damage [6]. Depending upon the number of joints assessed, the JADAS is scored on a continuous scale from 0–101 (71 joints), 0–57 (27 joints), or 0–40 (10 joints) and has good construct validity and responsiveness [6]. Changes in JADAS score are able to classify patients according to ACR Pedi response and have excellent ability to predict flare and inactive disease [34]. Recently, the “JADAS3,” which excludes ESR and avoids the necessity of a blood sample, was found to correlate with individual measures of disease activity as well as the original JADAS [58].

With improved success in the treatment of JIA, criteria for MDA, inactive disease, and remission have been defined. MDA is intended to define a state between high disease activity and remission that is acceptable to the physician and patient [42]; however, there is no data on the prognostic significance of maintaining this state. The MDA definitions have been validated in independent samples [42]. Similarly, the measurement properties of the Wallace criteria for inactive disease, remission on medication, and remission off medication have been assessed on data from 3 independent JIA clinics [43].

Systemic JIA (SJIA) differs from the other subtypes in that it is characterized by fever, rash, serositis, organomegaly, and lymphadenopathy, and these features must be considered in a disease activity measure. Batthish and colleagues have begun to develop an SJIA-specific disease activity measure using patient and parent interviews [48] and Delphi survey of health professionals to generate items [59]. The top items included fever, rash, increased CRP and ESR, and requirement for increasing medications. From here, the goal is to determine the best and most parsimonious list of items. At present, the ACR core set specifies the absence of spiking fever (≤38°C during past week) [2], and the definition of inactive disease requires absence of SJIA symptoms [15] as an additional requirement for SJIA children.

### 5.3. Disease Activity Measures in JSLE

Several disease activity measures initially developed in adult SLE patients have been found to be sensitive to change in JSLE [60]. The SLEDAI [35], British Isles lupus assessment group index (BILAG) [36], systemic lupus activity measure (SLAM) [37], and ECLAM [38] consist of many attributes grouped into 8–10 organ systems. The SLEDAI and SLAM are scored by weighting and summing of each attribute to give a total score. There are several rules for scoring the ECLAM to give an integer between 0 and 10. The BILAG uses an alphabetical rating system for each organ system, although numerical conversion schemes have been suggested [60]. Of the measures described, the SLEDAI and SLAM are the most user-friendly, and the ECLAM appears to be the most sensitive to change in JSLE [61]. The BILAG is complicated and takes longer to score.

### 5.4. Disease Activity Measures in JDM

Rider et al. have recently published a very comprehensive review of all the disease activity measures available for JDM, including their psychometric properties and utility for both clinical and research purposes [62]. The core sets for JDM include a measure of myositis activity assessed either with MMT or the childhood myositis assessment scale (CMAS). A modified MMT of 8 proximal, distal, and axial muscle groups tested unilaterally is scored from 0 to 80, and was found to closely approximate a total MMT score of 26 muscle groups tested bilaterally and is more feasible for use in the clinic setting as it takes less than 5 minutes to perform [63]. The CMAS uses 14 maneuvers (score 0–52) to measure muscle strength, physical function, and endurance and has excellent measurement properties [46]. The IMACS cores set considers the CMAS a measure of physical function, along with the CHAQ [12]. Levels of muscle enzymes are included in the IMACS core set as they are felt to be clinically useful but not in the PRINTO core set because they were found to correlate poorly with myositis activity in a clinical trial setting [14]. Global disease activity in JDM is measured with the DAS and the MDAAT. The DAS assesses muscle, cutaneous, and vasculopathic features of JDM [39], while the MDAAT includes extramuscular manifestations and assesses disease activity in 7 organ systems [40]. The cutaneous assessment tool (CAT) assesses the full range of cutaneous manifestations of JDM and provides both skin disease activity and skin damage scores [41].

Recently, criteria have been proposed for inactive disease in JDM (on or off therapy), requiring 3 or more of (1) CPK ≤ 150 U/I, (2) CMAS ≥ 48, (3) MMT ≥ 78, and (4) physician global assessment ≤0.2 [44]. These criteria should be externally validated prior to use in the clinical or research setting [64].

## 6. Conclusions

Over the last 20 years there has been an explosion of disease activity measures in paediatric rheumatology. The development of these tools is important as we aim to investigate and compare therapies for JIA, JSLE, and JDM. Challenges remain in determining which disease activity measures are optimal for use in the clinic and research settings. We are still working towards incorporating patient perspectives to obtain a comprehensive assessment of health in paediatric rheumatology patients.

## Figures and Tables

**Table 1:** Steps in the development of disease activity measures (adapted from [17, 24, 26, 27]).

Step	Comments
Statement of purpose of measure	Should be explicitly stated a priori
Methods of item generation	From the literature, expert opinion, patient opinion, consensus methods Item list should be comprehensive
Method to identify number of factors or constructs	For example, principal component and factor analysis
Derivation sample	Across spectrum of disease activity, academic and community clinics, ideally *n* = 200
Clinicians involved	Clinicians deriving criteria should be different than those providing cases/controls
Definition of gold standard	Validated set of criteria or expert opinions
Technique for selection of final set of items	For example, sensitivity/specificity, ROC curve, and regression models
Assessment of psychometric properties	Feasibility, face validity, content validity, reliability, construct validity, and responsiveness
External validation of criteria set	Distinct validation sample

**Table 2:** Definitions of domains of measurement properties, adapted from COSMIN taxonomy [28].

Domain	Measurement property	Definition
Reliability	Internal consistency	The degree of interrelatedness among items
	Reliability	The proportion of the total variance in measurements which is due to “true” differences among patients
	Measurement error	The systematic and random errors of a patient's score that is not attributed to true changes in the construct being measured

Validity	Content validity	The degree to which the content of an instrument is an adequate reflection of the construct to be measured
	Face validity	The degree to which the items look to be an adequate reflection of the construct to be measured
	Construct validity	The degree to which the scores of an instrument are consistent with hypotheses (e.g., relationships to scores of other instruments) based on the assumption that the instrument validly measures the construct to be measured
	Criterion validity	The degree to which the scores of an instrument are an adequate reflection of a “gold standard”

Responsiveness		The ability of an instrument to detect change over time in the construct to be measured

Interpretability		The degree to which one can assign qualitative meaning to an instrument's quantitative scores or change in scores

**Table 3:** Summary of disease activity measures for juvenile idiopathic arthritis (JIA), juvenile systemic lupus erythematosus (JSLE), and juvenile dermatomyositis (JDM).

	JIA	JSLE	JDM
Relative disease activity measures			
Core response variables (CRV)	ACR [10]: (1) Physician global assessment of disease activity (2) Parent/patient assessment of overall wellbeing (3) Functional ability (CHAQ) (4) Number of joints with active arthritis (5) Number of joints with limited range of motion (6) ESR For SJIA patients: absence of spiking fever (≤38°C during past week)	PRINTO [13]: (1) Physician global assessment of disease activity (2) Parent/patient assessment of overall wellbeing (3) Renal involvement: 24-hour proteinuria (4) Global disease activity tool (SLEDAI, ECLAM, or SLAM) (5) HRQL (CHQ)	PRINTO [14]: (1) Physician global assessment of disease activity (2) Parent/patient assessment of overall wellbeing (3) Muscle strength (CMAS, MMT) (4) Functional ability (CHAQ) (5) Global disease activity tool (DAS, MDAAT) (6) HRQL (CHQ) IMACS [12]: (1) Physician global assessment of disease activity (2) Muscle strength (MMT) (3) Functional ability (CHAQ, CMAS) (4) Muscle enzymes (≥2 of CPK, aldolase LDH, AST, ALT) (5) Extramuscular disease (MDAAT)
Definition of improvement	≥30% improvement from baseline in 3 of 6 CRVs, with ≤1 CRV worsening by >30% [10, 29]	≥50% improvement from baseline in 2 of 5 CRVs, with ≤1 CRV worsening by >30% [30]	PRINTO [31]: ≥20% improvement in 3 of 6 CRVs, with ≤2 worsening by ≥20% (muscle strength excluded) IMACS [32]: ≥15% improvement in muscle strength and physical function, ≥20% physician and patient global and extramuscular assessments, ≥30% improvement in muscle enzymes
Definition of flare	Worsening of 2 CRV by ≥40% without improvement in >1 CRV by ≥30% [33]		

Absolute disease activity measures			
Global disease activity tools	JADAS [6, 34]: (1) Physician global assessment of disease activity (2) Parent/patient global assessment of wellbeing (3) Count of joints with active disease (4) ESR	SLEDAI [35] BILAG [36] SLAM [37] ECLAM [38]	DAS [39] MDAAT [40] CAT [41]
Minimal disease activity (MDA)	MDA [42]: (1) Oligoarthritis: physician global assessment ≤2.5 cm and swollen joint count of 0 (2) Polyarthritis: physician global assessment ≤3.4 cm, parent global assessment ≤2.1 cm, and swollen joint count ≤1		
Inactive disease/remission	Wallace criteria [15, 43] *Inactive disease:* (1) No joints with active arthritis (2) No fever, rash, serositis, splenomegaly, or generalized lymphadenopathy attributable to JIA (3) No active uveitis (4) Normal ESR and/or CRP		PRINTO [44]: Inactive disease on or off medication, ≥3/4 of: (1) CPK ≤ 150 (2) CMAS ≥ 48 (3) MMT ≥ 78 (4) Physician global assessment ≤ 0.2
	(5) Physician global assessment indicates no disease activity *Clinical remission:* (1) On medication—criteria for inactive disease met for minimum 6 continuous months while patient on medication (2) Off medication—criteria for active disease met for minimum 12 continuous months while off all arthritis and uveitis medications		

ACR: American College of Rheumatology, CHAQ: child health assessment questionnaire, ESR: erythrocyte sedimentation rate, SJIA: systemic juvenile idiopathic arthritis, PRINTO: Pediatric Rheumatology International Trials Organization, SLEDAI: systemic lupus erythematosus disease activity index, BILAG: British Isles lupus assessment group index, SLAM: systemic lupus activity measure, ECLAM: European consensus lupus activity measurement, HRQL: health-related quality of life, CHQ: child health questionnaire, IMACS: International Myositis Assessment and Clinical Studies group, JADAS: juvenile arthritis disease activity score, MMT: manual muscle testing, CMAS: childhood myositis assessment scale, DAS: disease activity score, MDAAT: myositis disease activity assessment tool, and CAT: cutaneous assessment tool.

## Abbreviations

JIA: Juvenile idiopathic arthritis
JSLE: Juvenile systemic lupus erythematosus
JDM: Juvenile dermatomyositis
NGT: Nominal group technique
PRO: Patient-reported outcome.
